# Semi-Automated Processing of Harmonized Accelerometer and GPS Data in R: *AGPSR*

**DOI:** 10.3390/s25133883

**Published:** 2025-06-22

**Authors:** Nathan Tintle, Jieqi Tu, Anna Luong, Shina Min, Kiarri N. Kershaw, Ulf G. Bronas

**Affiliations:** 1 Department of Population Health Nursing Science, College of Nursing, University of Illinois Chicago, Chicago, IL 60612, USA; ntintle@uic.edu (N.T.); aluong5@uic.edu (A.L.); 2 Department of Biostatistics, UIC School of Public Health, University of Illinois–Chicago, Chicago, IL 60612, USA; jtu22@uic.edu; 3 School of Nursing, Columbia University, New York, NY 10032, USA; sm5567@cumc.columbia.edu; 4 Department of Preventive Medicine, Feinberg School of Medicine, Northwestern University, Chicago, IL 60611, USA; k-kershaw@northwestern.edu; 5 Division of Rehabilitation & Regenerative Medicine, Columbia University Irving Medical Center, New York, NY 10032, USA

**Keywords:** sensor data, software tools, data processing, data integration

## Abstract

The use of sensors in mobility, health, and place research is rapidly increasing, with the promise of objective quantification of human behavior patterns and the ability to understand complex patterns of human behavior in ways that were previously impossible. However, there is now a critical bottleneck in managing the resulting influx of sensor data. In particular, limited software pipelines exist that both clean and harmonize global positioning system (GPS) and accelerometer data into a single dataset that integrates information about time, space, and movement. Other approaches are costly due to proprietary software, personnel time, and being prone to human errors. To address this gap, we have created the open source *AGPSR* pipeline in the R software environment, combining GPS and accelerometer data to yield simultaneous information on location and activity status. Demonstrations of the pipeline and validity data are presented. The AGPSR software (Ver 1.0) is now available for researchers to use in studies involving simultaneous GPS and accelerometer wear.

## 1. Introduction

Sensor-based research in mobility, health, and place is becoming the norm, as researchers leverage technological advances to generate objective data to quantify human behavioral patterns [1]. The promise of such sensor-based research is large, as continuously and objectively measured behavioral patterns have the potential to catalyze massive breakthroughs in our understanding of the human condition. Recent reviews [1] provide important summaries of the novel concepts and terminology that have emerged within the last decade in this rapidly advancing field. However, these rapid improvements in the use of sensor-based data collection have created significant challenges in data processing. Chief among the challenges and opportunities presented by sensor-based research are issues of data quality, pre-processing, and harmonization [2]. These challenges and opportunities are particularly important when more than one sensor is used simultaneously, leading to cross-device data harmonization as a necessary step to spark the most significant breakthroughs in understanding human behavioral patterns. Advances in sensor-based research have further led to a rapidly expanding number of available choices of sensors, which vary considerably in both data quality and quantity generated [3]. The vast number of available devices and the large volume of data generated by these devices require careful consideration of study design, including participant training and device use fidelity, as well as a deep and clear understanding of device limitations (both systematic and random). These issues can be partially resolved after device use concludes, through high quality pre-processing, cleaning, and data integration techniques [1].

Importantly, despite the dizzying number of available sensors, there is a significant lag in the development of freely available (vs. proprietary and device-specific) and well-documented software. This lag creates a bottleneck in data processing and reduces the ability to combine data from various sensor-based technologies. Two of the most common passive, sensor-based device wearables in use today in mobility, health, and place studies are accelerometers (to measure activity) and global positioning system (GPS) loggers (to measure location) [4]. Moreover, while *R* continues to be one of the most widely used statistical programming languages globally due to its open-source, user-developed, and flexible framework [5], there are surprising limitations in existing R software options available for pre-processing and harmonizing of simultaneously collected accelerometer and GPS logger data, leading to substantial limitations in researchers’ ability to draw simultaneous conclusions about human activity-location patterns.

Historically, the interpretation of accelerometer data has been time-intensive and prone to the introduction of systematic and random errors when using human-centered data pre-processing protocols that leverage non-automatable, proprietary technology (e.g., classifying movement patterns based on visual interpretations of graphs in proprietary software like *ActiLife*^®^ [6]). In recent years, there has been a significant push to make accelerometer data pre-processing open-source and automated. These steps are important to improve data quality, to reduce potential bias, and to reduce the cost of human labor, while also improving accessibility and reproducibility [7]. Despite this effort, there are still substantial limitations in available R packages. For example, some existing accelerometer packages focus on sleep and subsequently do not attempt to classify or quantify movement patterns (e.g., minutes of moderate-to-vigorous activity, sedentary time, and bouts of activity, among many others). Other R packages for accelerometer data provide limited options for considering accelerometer wear location (e.g., wrist only vs. waist) [8,9,10].

Similarly, despite their dramatic rise in popularity [4], there continue to be substantial limitations with regards to automated, open-source data processing pipelines for GPS data. There are limitations when harmonizing GPS and accelerometer data outside of proprietary and selected sensor-based technologies, including processes, which limit choices and reduce data processing for research in health and place. Additionally, one of the most popular software tools (e.g., Physical Activity Location Measurement System [PALMS] [11]) is no longer supported, and some recently developed methodologies do not have published software [4,12,13,14]. Unfortunately, these limitations are exasperating because so few studies simultaneously gather data with both accelerometers and GPS loggers. For example, in a recent systematic literature review of mobility studies in older adults, only 1 out of 28 studies used both GPS and accelerometers [15]. This lack of processing options significantly limits our understanding of activity space influence on health and behavior.

We do not know of a currently available, open-source R data pipeline that simultaneously cleans and harmonizes accelerometer and GPS logger data over time and that simultaneously (1) provides information on both location and activity status, (2) harmonizes by daytime, and (3) addresses the limitations noted above. As part of an ongoing study [16], we needed to clean and harmonize GPS logger and accelerometer data and integrate these data into a larger workflow. Given the dearth of existing software options, we developed a customized R workflow, which we introduce in this paper. The accelerometer and GPS cleaning and harmonization pipeline for R (*AGPSR*) is an open-source R framework for the semi-automated, harmonized processing of accelerometer and GPS logger data. It is ready for high-throughput data processing, yielding data appropriate for ecological and other related study designs. Functionality provides user flexibility in decisions about data interpretation, cleaning, and imputation. Below, we explain the methodology, illustrate method validity, and summarize the basic functionality of the approach.

## 2. Methods

We begin by describing the current accelerometer and GPS sensor technology used in the Everyday Experiences and Environments (E3) study: a study to understand how environment influences individual diet and physical activity choices. The primary objective of the study is to address environmental exposures and provide a test of activity-space environment explanations for between- and within-person diet and physical activity (PA) variations during mid-life. The central hypothesis is that activity-space environmental exposures contribute to both between- and within-person variations in dietary and PA behaviors and strongly influence these behaviors. A key part of the study is to have participants wear sensors (GPS and actigraph) daily for three weeks and to investigate associations of participant wear patterns between these two devices. Four hundred participants aged 40–64 in Chicago, USA, were enrolled. A full description of the study and participant characteristics are provided elsewhere [16].

While *AGPSR* is designed to be a flexible R software framework, the initial development and testing of *AGPSR* has occurred within the E3 study, and so current features and limitations, as well as validity testing for *AGPSR*, have occurred within E3, making it important to understand the E3 study context.

### 2.1. E3 Study Protocols

#### 2.1.1. Accelerometer

The University of Illinois Chicago Institutional Review Board (IRB# 2016-0630) approved the E3 study, and all participants provided informed consent prior to any data collection procedures. The E3 study uses the *ActiGraph* wGT3X-BT (Actigraph LLC, Pensacola, FL, USA): a widely used physical activity monitor that continuously captures raw acceleration data using a tri-axial micro electromechanical system (MEMS) accelerometer [17]. All participants in E3 met with an E3 research team member and were provided verbal instructions and training on wearing the *ActiGraph* wGT3X-BT. All E3 participants wore the *ActiGraph* for 21 days around their waist, over thin clothing or over their skin, as this placement has been shown to capture more accurate data [18]. Participants were instructed to wear the *ActiGraph* continuously except when sleeping, bathing, showering, or swimming. Bluetooth wireless capabilities of the *ActiGraph* were disabled to allow for an estimated 24-day battery life with 30 Hz sample rate. Participants returned the *ActiGraph* by mail at the end of the 21-day study period. After *ActiGraph* receipt, researchers downloaded relevant files (e.g., *.gt3x files) for processing. *Actigraphs* were set to sample at a one-minute frequency in the E3 study.

#### 2.1.2. Global Positioning System (GPS)

E3 participants wore the Qstarz BT-Q1000XT Bluetooth Data Logger Global Positioning System (GPS) Receiver (Qstarz, Taipei, Taiwan), a device that tracks their navigation and travel log [19]. Participants were instructed to wear the GPS device throughout the day, except when bathing, showering, or swimming, and to ensure that the location logger charged during sleep time (participants were provided with GPS device charger). The GPS device was worn on a waistband alongside the accelerometer. The Qstarz BT-Q1000XT Bluetooth Data Logger GPS Receiver adopts high sensitivity—165 dBm and 66-channel tracking—to record participants’ daily travel activities and locations (such as grocery store, parks, gyms), using Google Earth geographical data. The typical battery operation time is 42 h under default settings. After the participant wore the GPS for 21 days, the GPS receiver was returned to research staff, and records were then downloaded to a computer, converted into raw files, which included details on their locations throughout wear time (for each minute of the day), and saved for further analysis.

### 2.2. AGPSR

**Figure 1:**
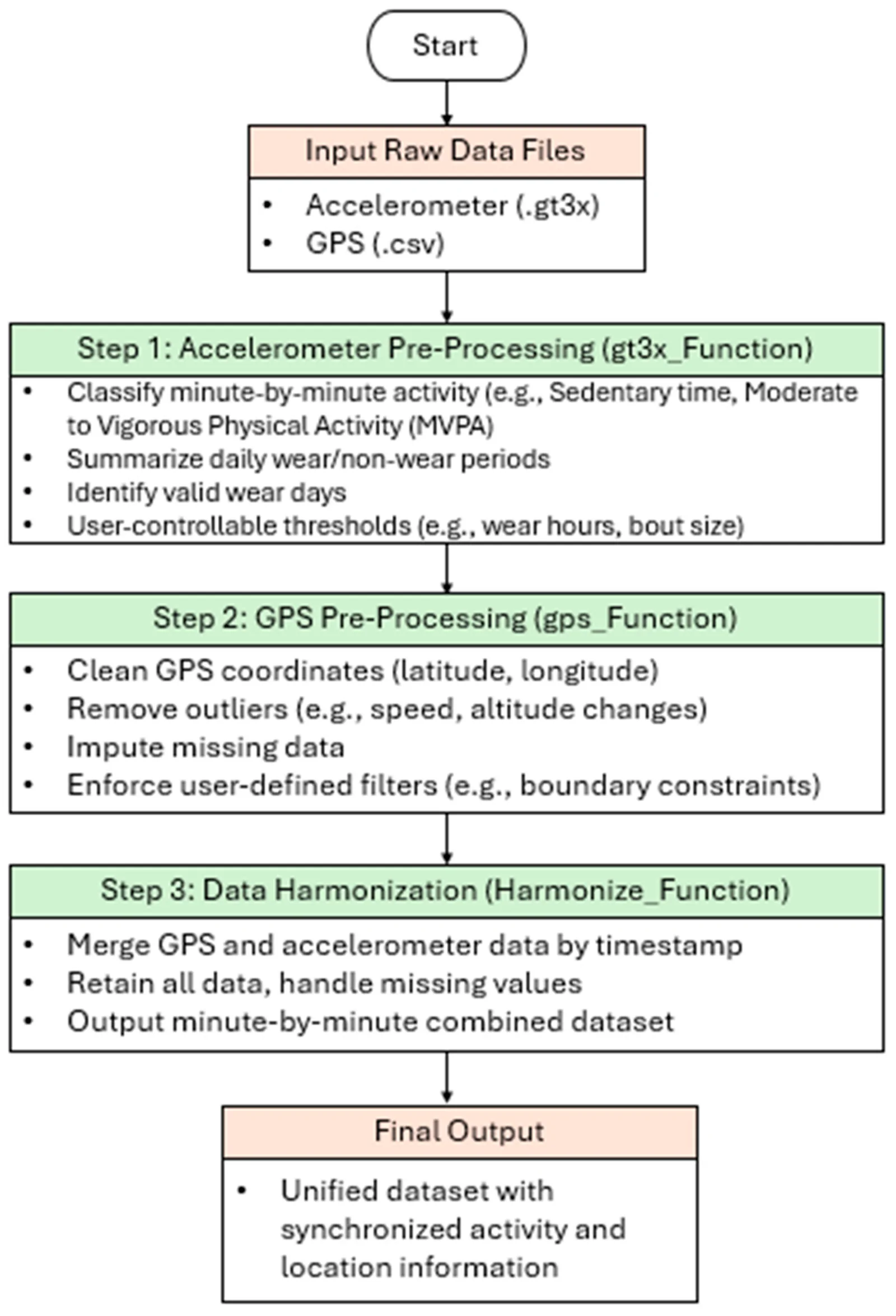
Overview of the AGSPR workflow.

AGPSR is a three-part R pipeline that (1) pre-processes and cleans accelerometer gt3x files, (2) pre-processes and cleans GPS logger comma-separated value (csv) files, and (3) harmonizes accelerometer and GPS logger files. Each of the three parts of AGPSR is coded as a separate R function, with clearly defined input file types and user-controllable parameters. AGPSR functions are provided on a freely available github page [20] along with additional documentation and example files. Detailed input values, methodological approaches, and outputs are also available in the supplemental materials. The following subsections provide a high-level overview of each of the three steps that make up AGPSR, while Figure 1 provides an overview of the workflow.

### 2.3. AGPSR Step 1. Accelerometer Data Pre-Processing via the gt3x_Function

Step 1 reads in a single *.*gt3x* file (e.g., a multi-day, single participant’s set of data) and outputs minute-by-minute classifications of movement type for each minute of a participant’s usable data (sedentary, low-light, high-light, light-moderate, high-moderate, or vigorous activity, as well as total moderate-vigorous activity [MVPA] for each minute of valid wear, based on Brond Counts [21], which include a frequency band-pass filter), as well as day-by-day summaries of non-wear and wear time, time of first/last wear each day, and decisions on whether the device was worn long enough each day for it to be considered a valid “wear day”. User-controllable parameters include the number of wear hours to be considered a valid day, bout sizes to help determine time of first/last wear each day, and the length of time of a midday window to detect non-wear time during the day, among other parameters. Step 1 is set to run on all files within a given directory to facilitate batch processing of files.

### 2.4. AGPSR Step 2. GPS Data Pre-Processing via the gps_Function

Step 2 reads in a single csv file generated by a GPS logger device. The input file is assumed to have a single row per minute and provides GPS coordinates (latitude and longitude) as well as time stamp information (date, time), encompassing all the GPS coordinates for a single participant for the course of the study. The intent of this function is that data has received little to no pre-processing cleaning elsewhere, though this is not a requirement. The output file is a single csv file that has cleaned and imputed latitude/longitude coordinates according to user-specified criteria. Data values are removed based on user-specified criteria, including speed, changes in height, and pre-specified geographic boundaries. Missing values are linear interpolated up to a user-specific time gap size.

### 2.5. AGPSR Step 3. Harmonized Accelerometer and GPS Data via the Harmonize_Function

Step 3 harmonizes the output from *AGPSR* steps 1 and 2 by merging data minute by minute. Specifically, this means identifying the day-time combinations that exist in both the accelerometer and GPS data and combining them (merging) in a one-to-one fashion. All data are retained, with missing data generated for either accelerometer or GPS data if needed. Resulting data can be further analyzed in downstream programs or used for various analyses.

### 2.6. Preliminary Validity and Software Demonstration

We randomly selected three E3 study participants from among the first 25 recruited to the study (participants are labeled A, B, and C). A single gt3x file (accelerometer) and a single csv file (GPS) were obtained from the sensor worn for each participant (see protocol details above). The entire three-step *AGPSR* function was run separately on each of the gt3x/csv file pairs. Accelerometer data were also processed using existing by-hand methods using the *ActiLife* software (see details above).

Preliminary data establishing validity of step 1 of *AGPSR* are provided by conducting pair-wise evaluations of a manual *ActiLife* scoring approach vs. *AGPSR* using a mix of qualitative and quantitative approaches (regression models/scatterplots; Bland–Altman plots). Input and output example data files are highlighted for all three steps of *AGPSR*. Random jitter is applied to all GPS coordinates to protect participant privacy. No other participant identifying information is provided.

### 2.7. Manual Actilife Scoring Approach

An alternative manual scoring approach using *Actilife* software was applied to the three selected participants. While details of the approach are described elsewhere [22], they are summarized here. Manual scoring (a) excludes the first and last day of data (potentially partial wear days and/or transport days), (b) excludes days with less than (estimated) 10 h of wear time, (c) identifies initial per-day wear time (e.g., first awake and wearing device) to the nearest quarter hour, and (d) identifies last per-day wear time (e.g., last awake and wearing device) to the nearest quarter hour. Identification of initial/last wear times per day were made to try to capture the first period of sustained activity. Data are then exported via a csv file for further analyses including summary measures of activity intensity, steps, raw acceleration, total movement, locomotion, and metabolic equivalent of task (MET) rates. *ActiLife* software setup, analysis, and scoring, when using this manual scoring approach, typically takes about 5–15 min per participant by a trained research assistant.

## 3. Results

### 3.1. AGSPR Step 1

**Figure 2:**
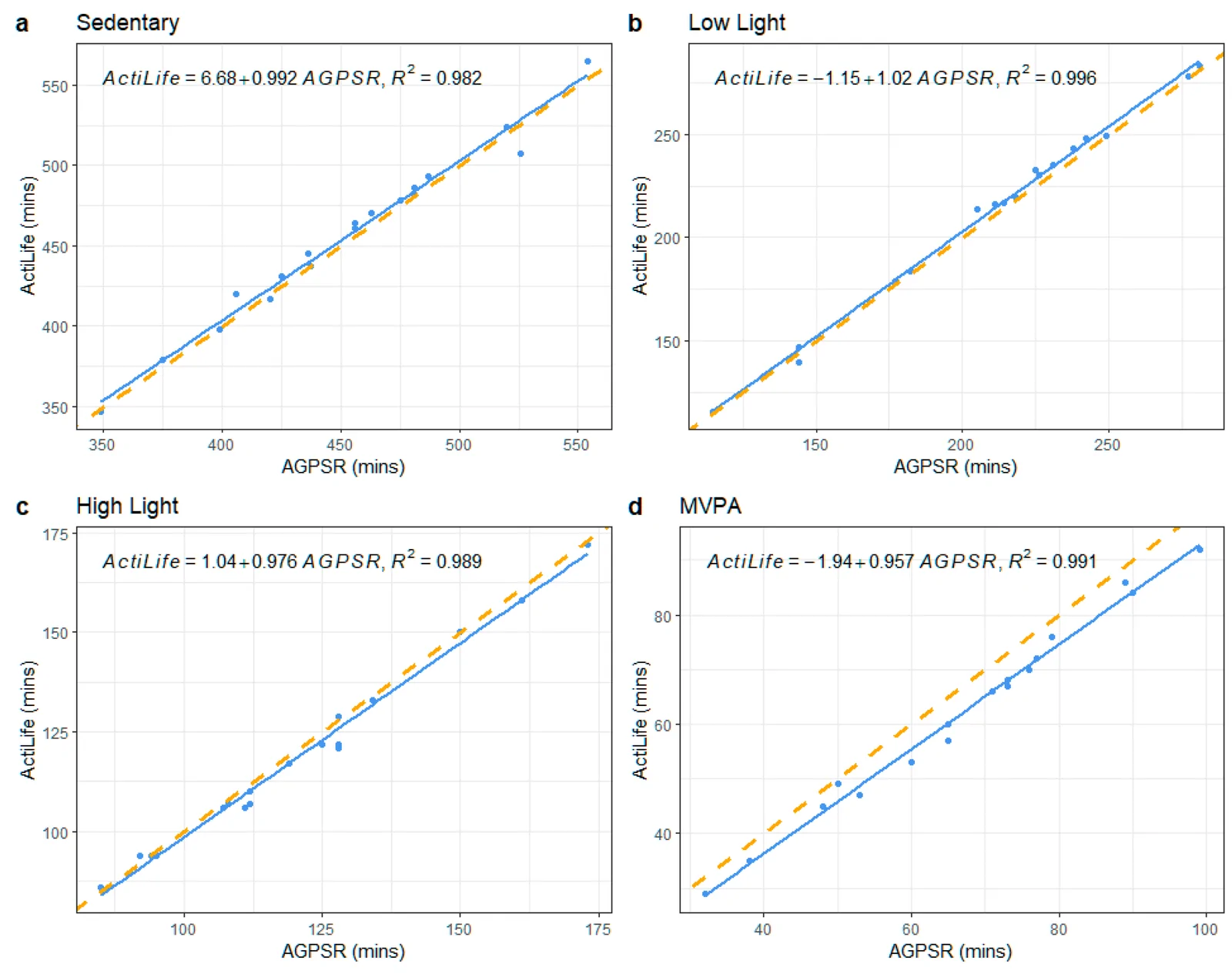
Scatterplots of *Actilife* results vs. AGPSR (participant B). Minutes of sedentary (**a**), low-light (**b**), high-light (**c**), and moderate-to-vigorous (MVPA) (**d**) activity are plotted based on the AGPSR pipeline vs. the *ActiLife* pipeline across a 21-day period for a single participant. The orange line is the line y = x, while the blue-dashed line is the best fit regression line. The association between the methods is high, as evidenced by the high R^2^ (over 0.98 in all cases). Furthermore, bias in the output from AGPSR is low (y-intercepts of the best fit line are close to zero, and slope coefficients are close to one) in all four cases.

**Figure 3:**
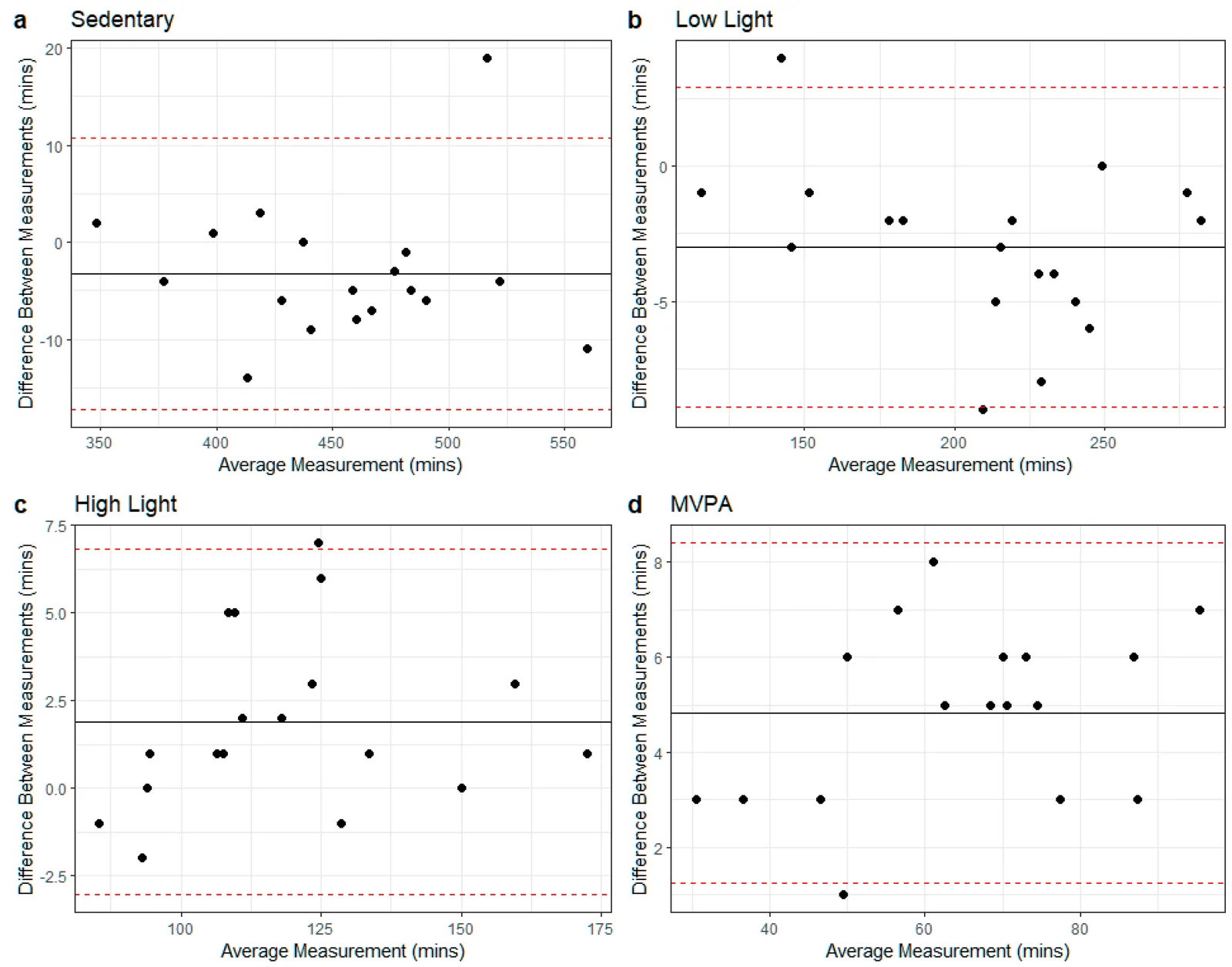
Bland–Altman plots of *ActiLife* vs. AGSPR (participant B). The plots highlight sedentary (**a**), low-light (**b**), high-light (**c**), and moderate-to-vigorous (MVPA) (**d**) activity for the AGPSR pipeline vs. the *ActiLife* pipeline across a 21-day period for a single participant. The average value of the two methods (AGPSR and *ActiLife*) on each day (*x*-axis) are plotted against the difference in the two measurements (*y*-axis), with lines representing the average difference (black) and mean ± 2 SD (red-dashed). With a loan exception (sedentary time on one day), the values show little to no outliers or drift.

*AGSPR* step 1 was run for participants A, B, and C. Figure 2 contains scatterplots for participant B comparing the minutes of sedentary, low-light, high-light, and MVPA for this participant across the 21-day period of the study (one dot per day). R^2^ values comparing the two methods are >0.98 in all cases, with little evidence of systematic bias (y-intercepts are close to zero, and slopes are close to 1) for both daily measures of light activity. There is a single outlier for sedentary activity that leads to bias in the comparison. This value (day) is described below (see next paragraph) and is completely explainable based on the user-controlled parameters for *AGPSR*. We also note that MVPA appears to have slightly more bias than other movement estimates, with *AGSPR* underestimating (relative to *ActiLife*) MVPA by approximately 2 min per day on average. Corresponding Bland–Altman plots are provided in Figure 3 and show little to no drift in movement estimates. Similar plots and results for participant B with the outlier, along with plots for participants A (with and without a single explainable outlier) and C, are provided in Supplemental Figures S1–S8.

Table 1 provides a side-by-side comparison of the results of the *ActiLife* pipeline and *AGPSR* step 1. Overall, we can see that the first/last wear minutes are very similar, which leads to similar estimates of wear time. The largest exception is May 14th (the outlier value for participant B; Supplemental Figures S1 and S2). This outlier is explained by a large difference in the “last wear minute” determination from *ActiLife* and *AGPSR*. We applied *AGPSR* using a very strict “no movement” rule, whereas the *ActiLife* protocol is less sensitive and allows for very small movements early in the day/at the end of the day to be excluded from wear time calculations. Supplemental Tables S1a and S1b are provided for participants A and C and yield similar results.

### 3.2. AGPSR Step 2

Table 2 is an example of the output from AGPSR step 2—GPS data cleaning with user-controllable data cleaning/imputation parameters. Table 2 illustrates minute-by-minute data on participant location (latitude and longitude), along with the participant’s height above sea-level at that point and GPS device-inferred speed based on distance traveled. Data shown are after user-defined data cleaning procedures have been applied (e.g., imputing missing data using linear interpolation, etc.).

**Figure 4:**
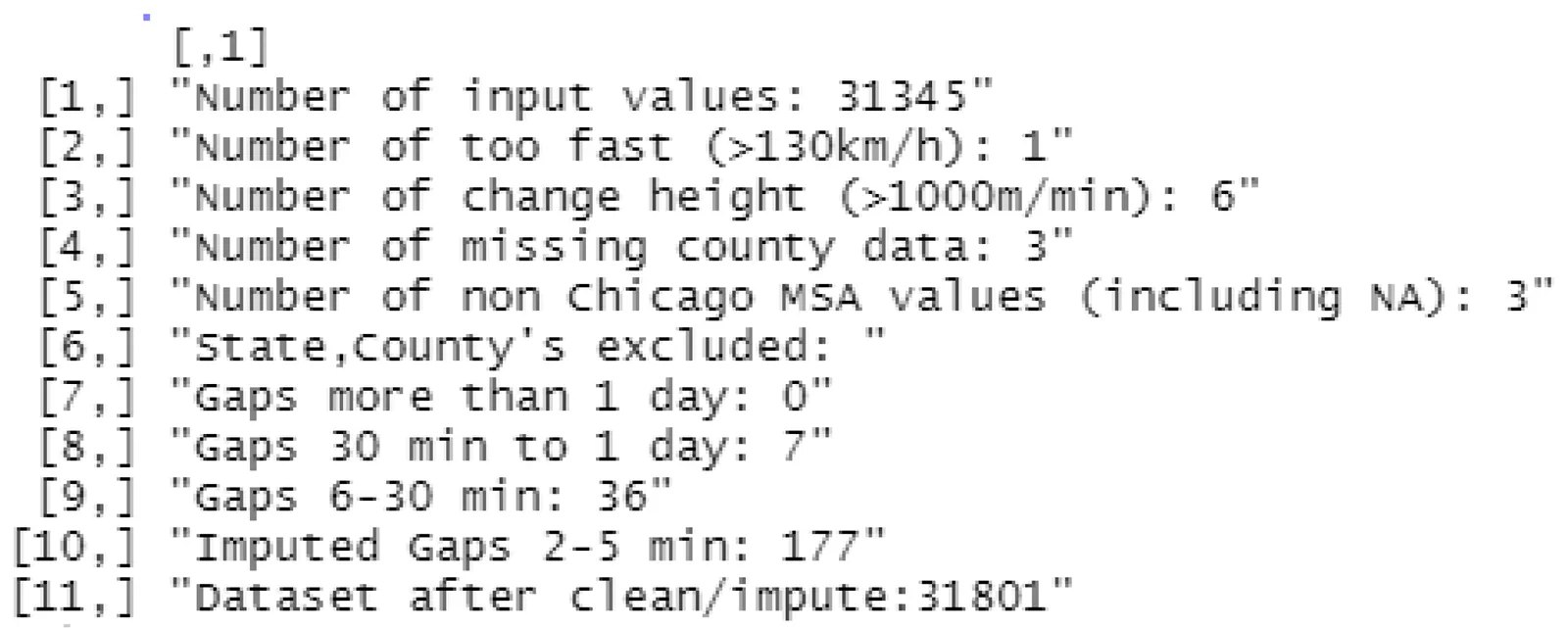
Output from applying AGPSR to GPS data from participant B.

Figure 4 provides summary output statistics from running step 2 of AGPSR using user-controllable parameters for speed, height changes, incorrect county data, and gap size, showing limited amounts of missing data, with 177 data values imputed.

### 3.3. AGPSR Step 3

Selected results from running AGPSR Step 3 are provided in Table 3. This step merges accelerometer and GPS data, providing minute-by-minute GPS location information and activity type classifications.

## 4. Discussion

Despite rapid growth in the use of sensor-based research, there are substantial gaps in user-friendly, open-source, modifiable software tools. In this manuscript, we present *AGPSR*, an open-source R framework for the simultaneous pre-processing, cleaning, and harmonizing of accelerometer and GPS data. The code is available on github, and sample applications of the software show excellent consistency with by-hand scoring from *ActiLife* software.

*AGPSR* represents a substantial step forward for data analysis needs by providing simultaneous information about a participant’s place and their activity level. This type of information is critical for catalyzing our understanding not only of where people spend their time or what kinds of activities comprise a person’s day but also the joint relationships between place and activity type.

*AGPSR* has many user-friendly, customizable features, including customizable features for filtering data based on speed/height changes, locations outside of a set area, and gap size to impute/interpolate, among others. We note that the use of minute-by-minute data here is merely only of convenience, reflecting the E3 study design. AGPSR does assume that input data files are measured on the same unit (e.g., each row in raw data files represents a unit of time), though units other than minutes are possible (e.g., seconds), with other customizable features scaled accordingly. We also note that while the demonstration of the software here is on human participants, other applications may be possible, though additional validity testing in these scenarios will be necessary [23].

There are some limitations worth noting. First, there are limitations of the software with regard to additional quality measures (e.g., Position Dilution of Precision [PDOP]), more sophisticated data harmonization (e.g., just accelerometer data missing or just GPS missing), additional accelerometer measures (activity intensity, steps), alternative filtering (e.g., Kalman [24]), imputation (beyond just linear interpolation), comparison with other proprietary methods, and batch submission. While these are all reasonable extensions, our approach here is twofold: (1) This software was developed for a single study with specific goals, and so efforts were made to provide the minimal set of features needed for the parent study while (2) providing an open-source, easily editable/modifiable workflow and script and allowing user customization. This approach is in line with that of the overarching R user community—namely, to provide frameworks on which others can innovate. In this case, many of the limitations above are easily addressed through additional lines of code, modified function calls, and other modifications. It is also possible that some features will be added in future software releases.

Another limitation is that of validity testing. We restricted initial validity testing to a comparison of a standard accelerometer processing pipeline with the new software. Overall, the results are very consistent, with minor differences generally explainable by user-customizable decisions. We note that the participants selected for validity testing did not have high levels of MVPA, and so additional comparisons may be warranted in this case. However, even though bias was potentially slightly higher, absolute levels of bias (~2 min/day) were small and systematic and, thus, may have limited impact on downstream analyses.

Finally, we note that there is a push to harmonize data across numerous other sensors or self-reported measures [15]. Future work will address further harmonization with other sources of activity and/or location information.

In conclusion, the AGSPR R package for GPS-accelerometer pre-processing, cleaning, and harmonization is freely available and ready for widespread use. Users are free to use the software as is, modify it for their own customized use, request feature additions, and test the software’s performance in alternative settings, and it provides a fully reproducible, automated and effect approach to data cleaning and harmonization. Ongoing work continues to explore additional features, robustness, and additional validity tests in other settings.

## Figures and Tables

**Table 1:** Day-by-day comparisons of AGPSR vs. *ActiLife* (participant B).

Date	First Wear Minute		Last Wear Minute		Non-Wear Time (Hours)		Wear Time (Hours)		Decision on Inclusion		Reason for Exclusion	
	ActiLife	R	*ActiLife*	R	*ActiLife*	R	*ActiLife*	R	*ActiLife*	R	*ActiLife*	R
**4/28/2022**									Exclude	Exclude	1st day of study	1st day of study
**4/29/2022**	7:00 a.m.	6:55:00	9:45 p.m.	21:37:00	9.25	0	14.25	14.7	Include	Include		
**4/30/2022**	7:15 a.m.	7:15:00	10:30 p.m.	22:20:00	10.75	0.75	13.25	14.33	Include	Include		
**5/1/2022**	8:15 a.m.	8:08:00	10:00 p.m.	21:54:00	10.75	1.5	16	12.27		Include		
**5/2/2022**	7:15 a.m.	7:13:00	9:30 p.m.	21:26:00	9.75	0	14.5	14.22		Include		
**5/3/2022**	6:45 a.m.	6:50:00	10:00 p.m.	21:55:00	10.75	0	14.25	15.08		Include		
**5/4/2022**	7:00 a.m.	7:00:00	8:00 p.m.	20:04:00	11	0	14.75	13.07		Include		
**5/5/2022**	7:00 a.m.	6:55:00	9:15 p.m.	21:08:00	9.75	0	14.25	14.22		Include		
**5/6/2022**	6:45 a.m.	6:45:00	8:45 p.m.	20:42:00	10	0	14	13.95		Include		
**5/7/2022**	8:00 a.m.	8:01:00	9:15 p.m.	21:15:00	10.75	0.75	14.75	12.48		Include		
**5/8/2022**	8:00 a.m.	8:02:00	9:15 p.m.	21:15:00	10.75	0	14.5	13.22		Include		
**5/9/2022**	6:30 a.m.	6:35:00	9:15 p.m.	21:10:00	9.25	0	14.5	14.58		Include		
**5/10/2022**	6:45 a.m.	6:39:00	8:45 p.m.	20:45:00	10	0	14	14.1		Include		
**5/11/2022**	Excluded	16:43:00	Excluded	20:10:00	Excluded	0	<10	3.45	Exclude	Exclude	<10 h wear time	wear time < 10 h
**5/12/2022**	7:15 a.m.	7:12:00	10:15 p.m.	22:08:00	9	0	14.25	14.93		Include		
**5/13/2022**	7:15 a.m.	6:40:00	9:15 p.m.	21:14:00	10	0	13.25	14.57		Include		
**5/14/2022**	9:30 a.m.	9:28:00	8:15 p.m.	22:41:00	13.25	0	13.25	13.22		Include		
**5/15/2022**	8:45 a.m.	8:42:00	9:15 p.m.	21:16:00	11.5	1.5	13.25	11.07		Include		
**5/16/2022**	7:30 a.m.	7:29:00	9:30 p.m.	21:20:00	10	0	15.25	13.85		Include		
**5/17/2022**	6:30 a.m.	6:35:00	9:15 p.m.	21:17:00	9.25	0	13.75	14.7		Include		
**5/18/2022**	7:00 a.m.	7:04:00	9:00 p.m.	21:05:00	10	0.75	14	13.27		Include		
**5/19/2022**	Excluded	7:18:00	Excluded	23:57:00	Excluded	7.5	Excluded	9.15	Exclude	Exclude	<10 h wear time	wear time <10 h

Note: Data were also collected for 5/20/2022–5/24/2022; however, these data were excluded by both approaches because it was past day 21 of the study.

**Table 2:** Example of output data file from AGPSR step 2. Participant B. Randomly selected rows.

Local Date	Local Time	Latitude	North (N)/South (S)	Longitude	East (E)/West (W)	Height (M)	Speed (km/h)
…	…	…	…	…	…	…	…
2022/05/12	12:49:54	43.08822	N	90.06465	W	150.083	0.086
2022/05/12	12:50:54	43.08822	N	90.06465	W	150.263	0.120
2022/05/12	12:51:54	43.08822	N	90.06465	W	150.366	0.152
2022/05/12	12:52:54	43.08821	N	90.06464	W	150.411	0.041
2022/05/12	12:53:54	43.08821	N	90.06463	W	150.512	0.059
2022/05/12	12:54:54	43.08821	N	90.06463	W	150.527	0.061
2022/05/12	12:55:54	43.08821	N	90.06463	W	150.724	0.067
2022/05/12	12:56:54	43.0882	N	90.06463	W	150.776	0.116
2022/05/12	12:57:54	43.0882	N	90.06463	W	151.118	0.147
2022/05/12	12:58:54	43.0882	N	90.06461	W	150.992	0.229
2022/05/12	12:59:54	43.08819	N	90.06461	W	150.895	0.069
2022/05/12	13:00:54	43.08819	N	90.06461	W	150.981	0.152
2022/05/12	13:01:54	43.08819	N	90.06461	W	151.061	0.210
2022/05/12	13:02:54	43.08814	N	90.06454	W	150.809	0.690
2022/05/12	13:03:54	43.08809	N	90.06459	W	150.271	0.772
2022/05/12	13:04:54	43.08811	N	90.0646	W	151.155	0.897
2022/05/12	13:05:54	43.08804	N	90.06461	W	151.734	1.754
2022/05/12	13:06:54	43.08817	N	90.06452	W	151.621	1.309
2022/05/12	13:07:54	43.08825	N	90.06464	W	151.464	0.572
2022/05/12	13:08:54	43.08826	N	90.06464	W	151.070	0.515
2022/05/12	13:09:54	43.08827	N	90.06464	W	154.035	0.740
2022/05/12	13:10:54	43.08829	N	90.06466	W	157.247	0.571
2022/05/12	13:11:54	43.08829	N	90.06468	W	159.437	0.049
2022/05/12	13:12:54	43.08829	N	90.06467	W	159.374	0.043
2022/05/12	13:13:54	43.08828	N	90.06467	W	159.315	0.137
…	…	…	…	…	…	…	…

**Table 3:** Example of output data file from AGPSR step 2. Participant B. Randomly selected rows.

Local Date	Local Time	Latitude	North (N)/South (S)	Longitude	East (E) or West (W)	Height (M)	Speed	Activity Type
…	…	…	…	…	…	…	…	…
2022/05/12	12:49:54	42.8391	N	85.34715	W	160.264	0.149	Sedentary
2022/05/12	12:50:54	42.83911	N	85.34715	W	160.086	0.173	Sedentary
2022/05/12	12:51:54	42.83911	N	85.34714	W	160.123	0.157	Sedentary
2022/05/12	12:52:54	42.8391	N	85.34714	W	160.375	0.093	Sedentary
2022/05/12	12:53:54	42.8391	N	85.34713	W	160.338	0.065	Sedentary
2022/05/12	12:54:54	42.83909	N	85.34713	W	160.422	0.025	Sedentary
2022/05/12	12:55:54	42.8391	N	85.34712	W	160.350	0.031	Low light
2022/05/12	12:56:54	42.8391	N	85.34712	W	160.305	0.063	Low light
2022/05/12	12:57:54	42.8391	N	85.34712	W	159.964	0.035	Low light
2022/05/12	12:58:54	42.8391	N	85.34712	W	160.022	0.007	High light
2022/05/12	12:59:54	42.8391	N	85.34712	W	159.706	0.052	Sedentary
2022/05/12	13:00:54	42.8391	N	85.34712	W	159.438	0.043	Sedentary
2022/05/12	13:01:54	42.8391	N	85.34712	W	159.150	0.048	Sedentary
2022/05/12	13:02:54	42.8391	N	85.34712	W	158.740	0.005	Sedentary
2022/05/12	13:03:54	42.8391	N	85.34711	W	158.473	0.068	Sedentary
2022/05/12	13:04:54	42.8391	N	85.34712	W	158.068	0.024	Sedentary
2022/05/12	13:05:54	42.8391	N	85.34712	W	157.828	0.032	Sedentary
2022/05/12	13:06:54	42.8391	N	85.34712	W	157.755	0.010	Sedentary
2022/05/12	13:07:54	42.8391	N	85.34712	W	157.648	0.029	Sedentary
2022/05/12	13:08:54	42.8391	N	85.34712	W	157.797	0.014	Sedentary
2022/05/12	13:09:54	42.8391	N	85.34713	W	157.913	0.024	Sedentary
2022/05/12	13:10:54	42.8391	N	85.34713	W	158.087	0.038	Sedentary
2022/05/12	13:11:54	42.8391	N	85.34713	W	158.096	0.046	Sedentary
2022/05/12	13:12:54	42.8391	N	85.34713	W	158.145	0.035	Sedentary
2022/05/12	13:13:54	42.8391	N	85.34712	W	157.846	0.025	Sedentary
…	…	…	…	…	…	…	…	…

## Data Availability

The original contributions presented in this study are included in the article/Supplementary Material. Further inquiries can be directed to the corresponding author.

## Supplementary Materials

The following supporting information can be downloaded at https://www.mdpi.com/article/10.3390/s25133883/s1, Figure S1: Participant B—including outlier value; Figure S2: Participant B—with outlier; Figure S3: Participant A after removing a single explainable outlier; Figure S4: Participant A after removing a single explainable outlier; Figure S5: Participant A before removing a single explainable outlier; Figure S6: Participant A before removing a single explainable outlier; Figure S7: Participant C; Figure S8: Participant C; Table S1a: Side by Side Comparisons of Wake/Sleep/Non-wear Times- Participant A; Table S1b: Side by Side Comparisons of Wake/Sleep/Non-wear Times- Participant C.
