# Supplementary material for: Semi-Automated Processing of Harmonized Accelerometer and GPS Data in R: AGPSR
Source: Sensors (Basel). 2025 Jun 22;25(13):3883. doi: 10.3390/s25133883 (PMC12252245; doi:10.3390/s25133883)
Supplement: Supplementary file 1 [file sensors-25-03883-s001.zip › sensors-3592348-supplementary.pdf]

Supplemental Methods

AGPSR Step 1. Accelerometer pre-processing via gt3x_function

The *gt3x_function* takes in a single gt3x file (e.g., a multi-day, single participant's set of data), and outputs two data files: a csv file with minute-by-minute classifications of movement type for each minute of a participant's usable data, and a csv file with wake/sleep time, non-wear and wearing time on each day and decisions on whether to include this day into the study. User controllable parameters include:

(a) the directory containing all the gt3x files to process (data_directory),
(b) the number of wear hours required for a day to be considered valid (*valid_day_criteria*: default 10 hours),
(c) the minimum per-minute bout size detected at the start of the 24-hour day to be considered "first wear" (*wake_bout*: default 1 minute),
(d) the minimum per-minute bout size detected at the end of the 24-hour day to be considered "first wear" (*sleep_bout*: default 1 minute)
(e) the minimum amount of time with no detected movement, which is classified as midday non-wear time (*non_wear_chunk_min*: default 45 minutes),
(f) the earliest hour of the day to look for wear time (*start_hour*: default 4:00AM),
(g) the participant ID (participant_id: default P2E30001),
(h) the maximum number of consecutive days of data to include (days_include: default 21).

gt3x_function leverages existing R packages for additional functionality to process accelerometer data. Namely, the *activityCounts* and *SummarizedActigraphy* packages are used to convert activity counts data into activity classifications. Brond counts are used, and valid wear time activity is classified as: Sedentary, Low-light, High-light, Light-Moderate, High-Moderate or Vigorous activity, as well as total moderate-vigorous activity (MVPA) for each minute of valid wear. The first output datafile yields one row for each minute of valid wear with columns for date, time, activity counts and activity classification. The output datafile 2 summarizes wake time, sleep time, non-wear time (hours), sleep minus wake time (hours), wear time, and decision on inclusion on each day.

AGPSR Step 2. GPS data pre-processing via gps_function

The *gps_function* reads in a single csv file generated by a GPS logger device. The file is assumed to have a single row for each different minute, provides GPS coordinates (latitude and longitude) as well as time stamp information (date, time), and encompasses all the GPS coordinates for a single participant for the course of the study. The intent of the gps_function is that data has received little to no pre-processing cleaning elsewhere, though this is not a requirement. The output file is a single csv file which has been cleaned and imputed latitude/longitude coordinates according to user-specified criteria. User controllable parameters include:

(a) the maximum speed to include; values above this speed are made missing (maxspeed; default: 130 km/h),
(b) the maximum change in height over 1 minute to include; values above this are set to missing (maxhtchange: default 1000m),
(c) counties to include; all others are excluded (counties_list; user-specified list of counties in state,countyname format; NA includes all counties)

(d) the maximum gap size to impute using linear interpolation; remaining gap sizes are left as missing (gapsize; default = 5 minutes)

After running *gps_function,* the user will see output that provides summary data on how much data was eliminated based on user-controllable parameters (a)–(c), and how many values were imputed/gaps remaining that were too large impute (based on (d)).

AGPSR Step 3. Harmonized accelerometer and GPS data via harmonize_function

The *harmonize_function* is simply a wrapper function to merge the output *AGPSR* Steps 1 and 2 according to simple rules. The function takes in a single Step 1 output file and a single Step 2 output file and merges the files by minute. At this time, the function keeps all rows (minutes) from both datafiles, leaving missing data for the accelerometer or the GPS data as needed. Straightforward editing or post-processing data elimination can identify output data rows for deletion. The output file is a single csv with one row per minute, including both latitude/longitude and activity classification.

Supplemental Figure S1 – Participant B – including outlier value

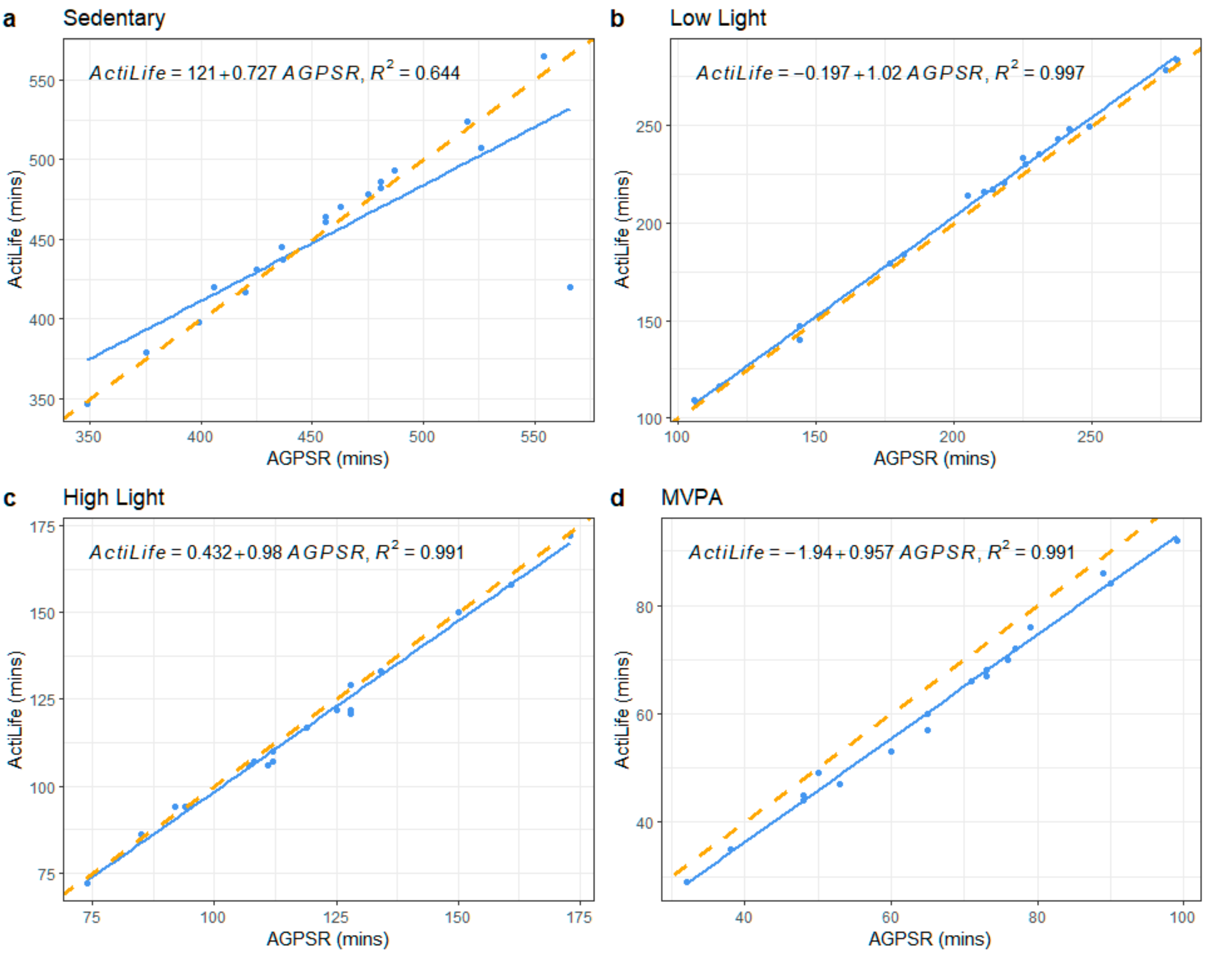

Supplemental Figure S2 – Participant B – with outlier

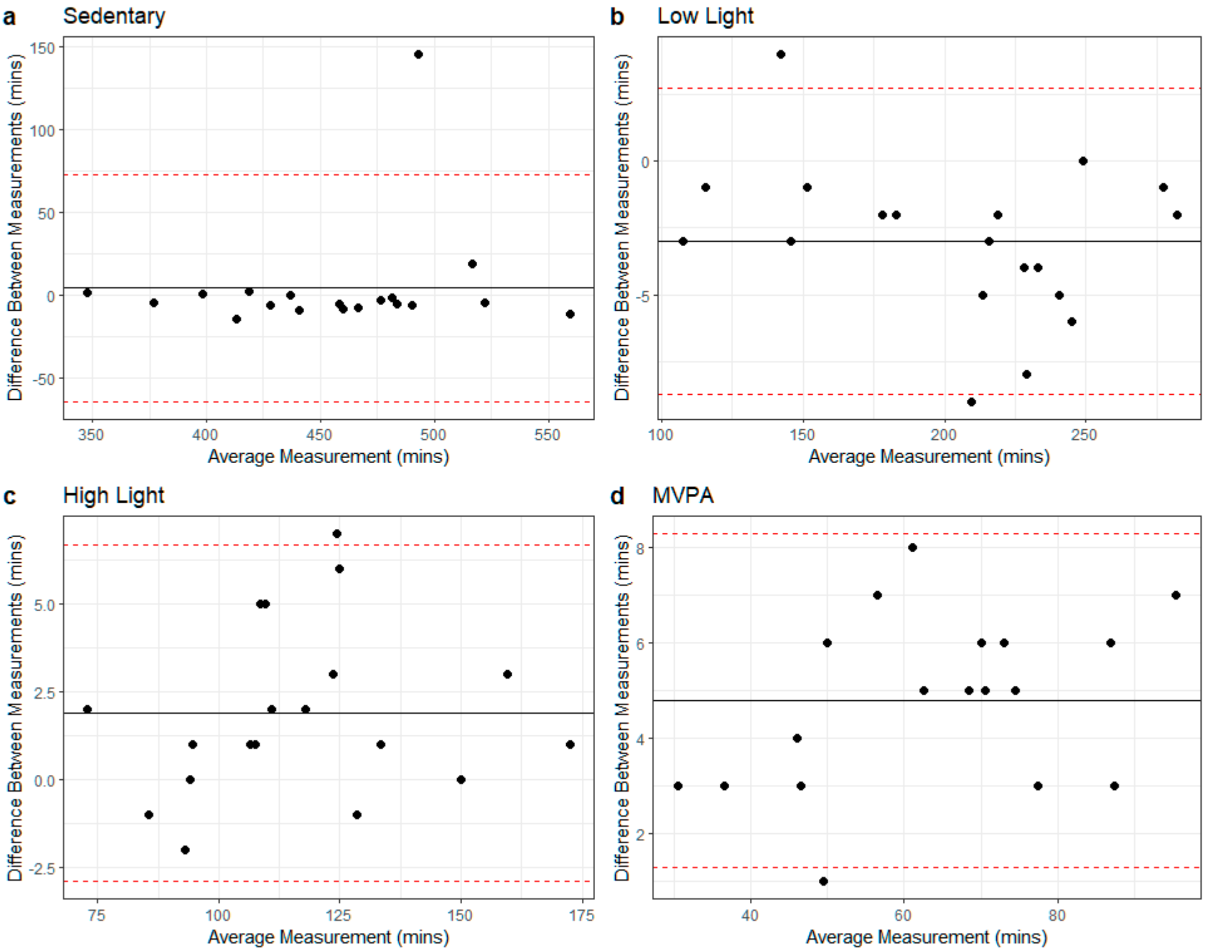

Supplemental Figure S3 – Participant A after removing a single explainable outlier

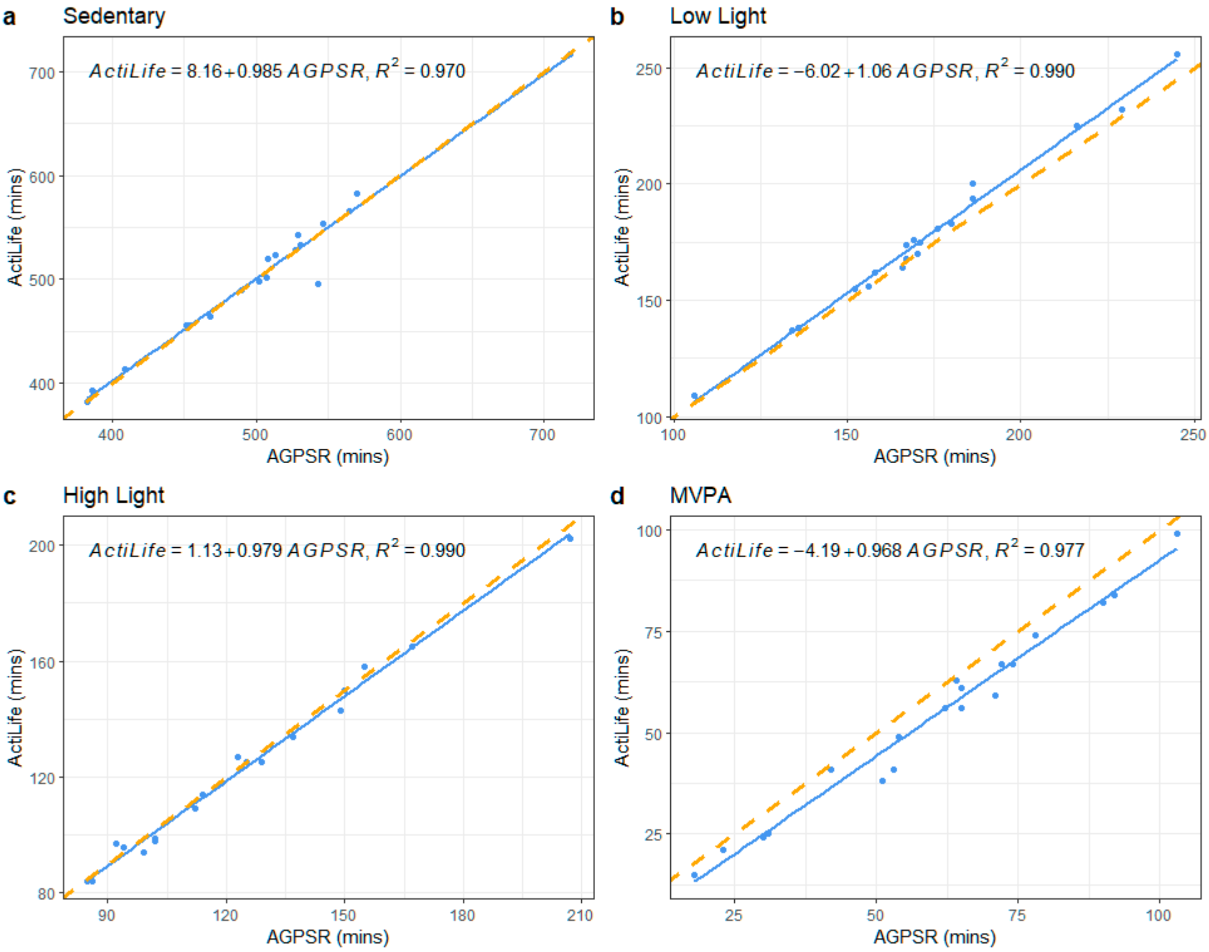

Figure S3: The single outlier that was removed is similar to the outlier for Participant B (see primary text).

Supplemental Figure S4 – Participant A after removing a single explainable outlier

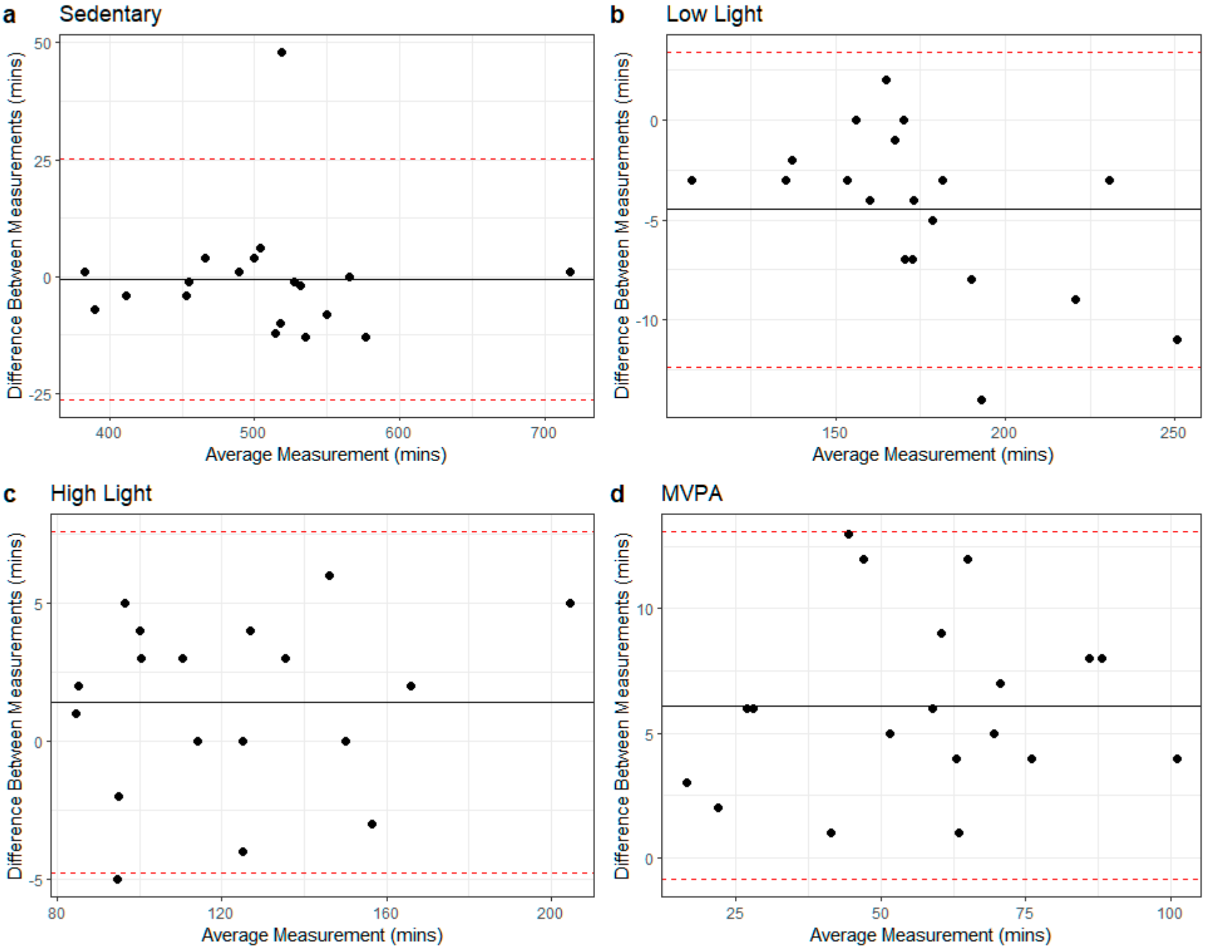

Figure S4: The single outlier that was removed is similar to the outlier for Participant B (see primary text).

Supplemental Figure S5 – Participant A before removing a single explainable outlier

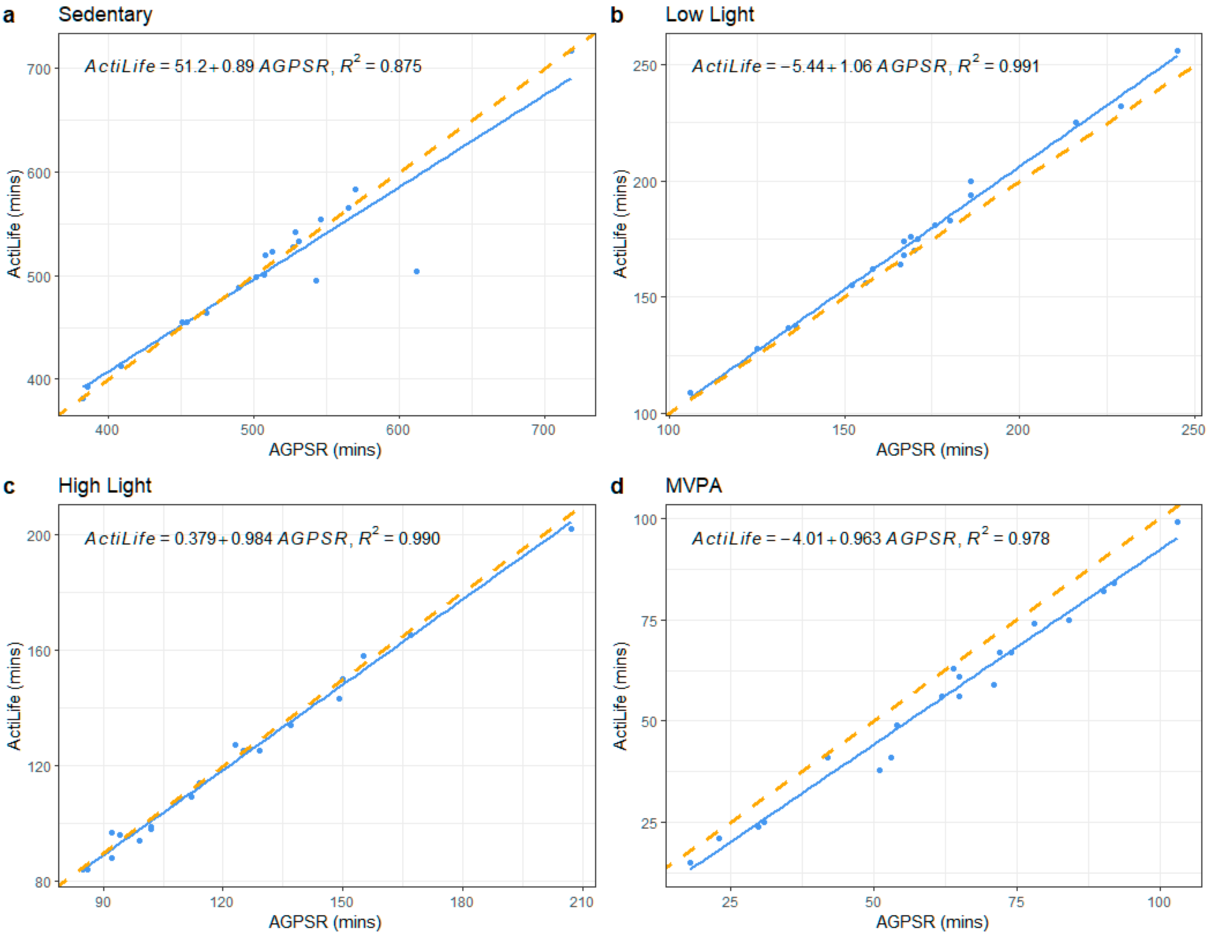

Supplemental Figure S6 – Participant A before removing a single explainable outlier

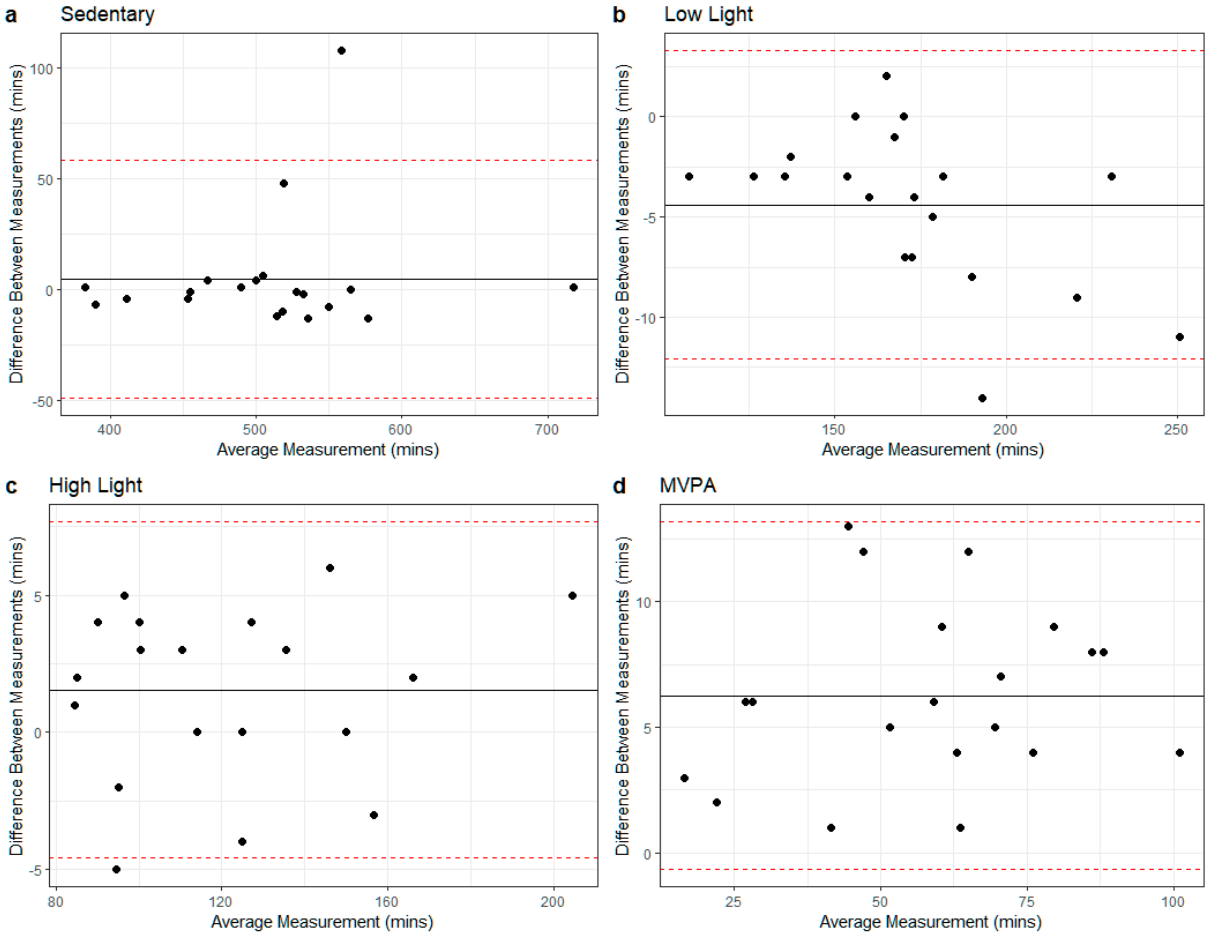

Supplemental Figure S7 – Participant C

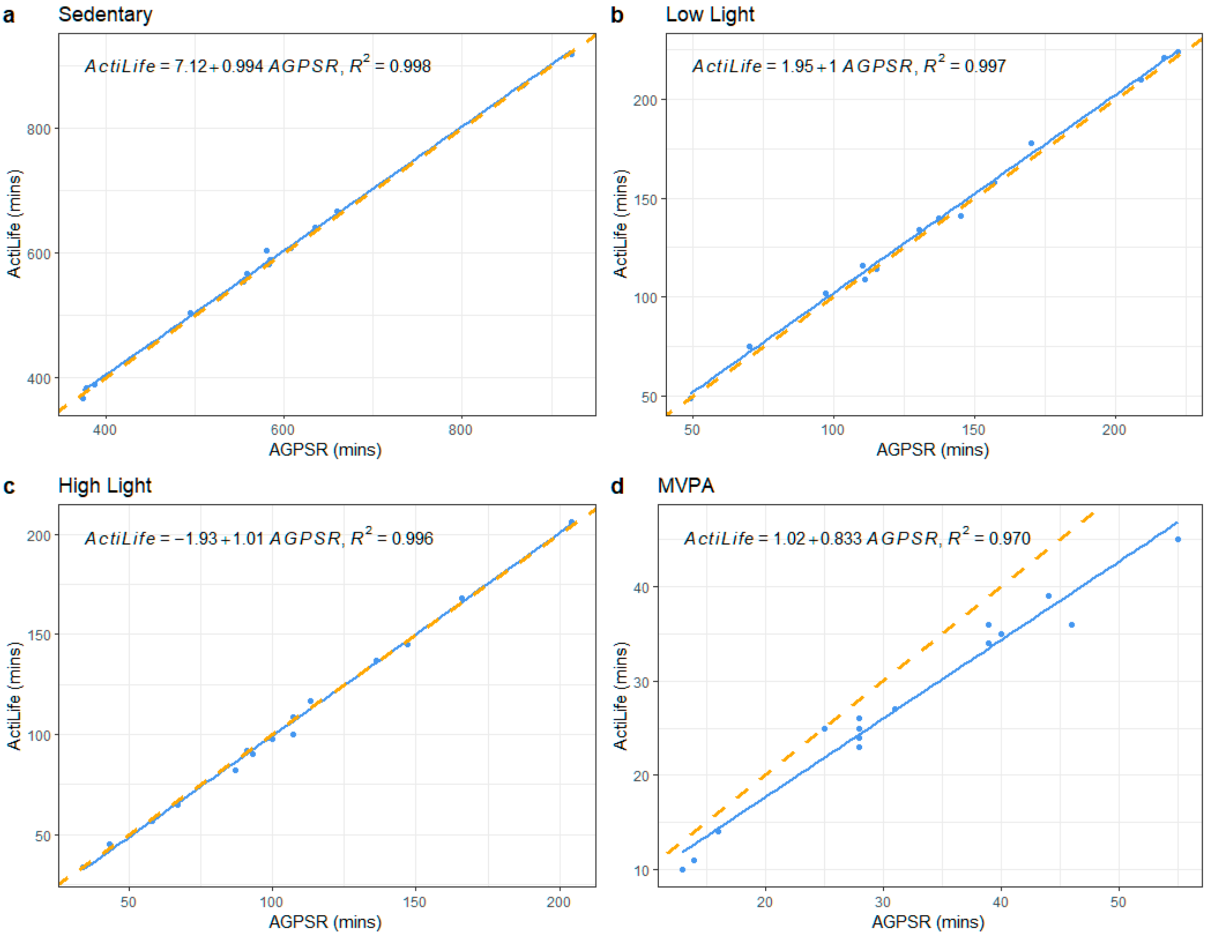

Supplemental Figure S8 – Participant C

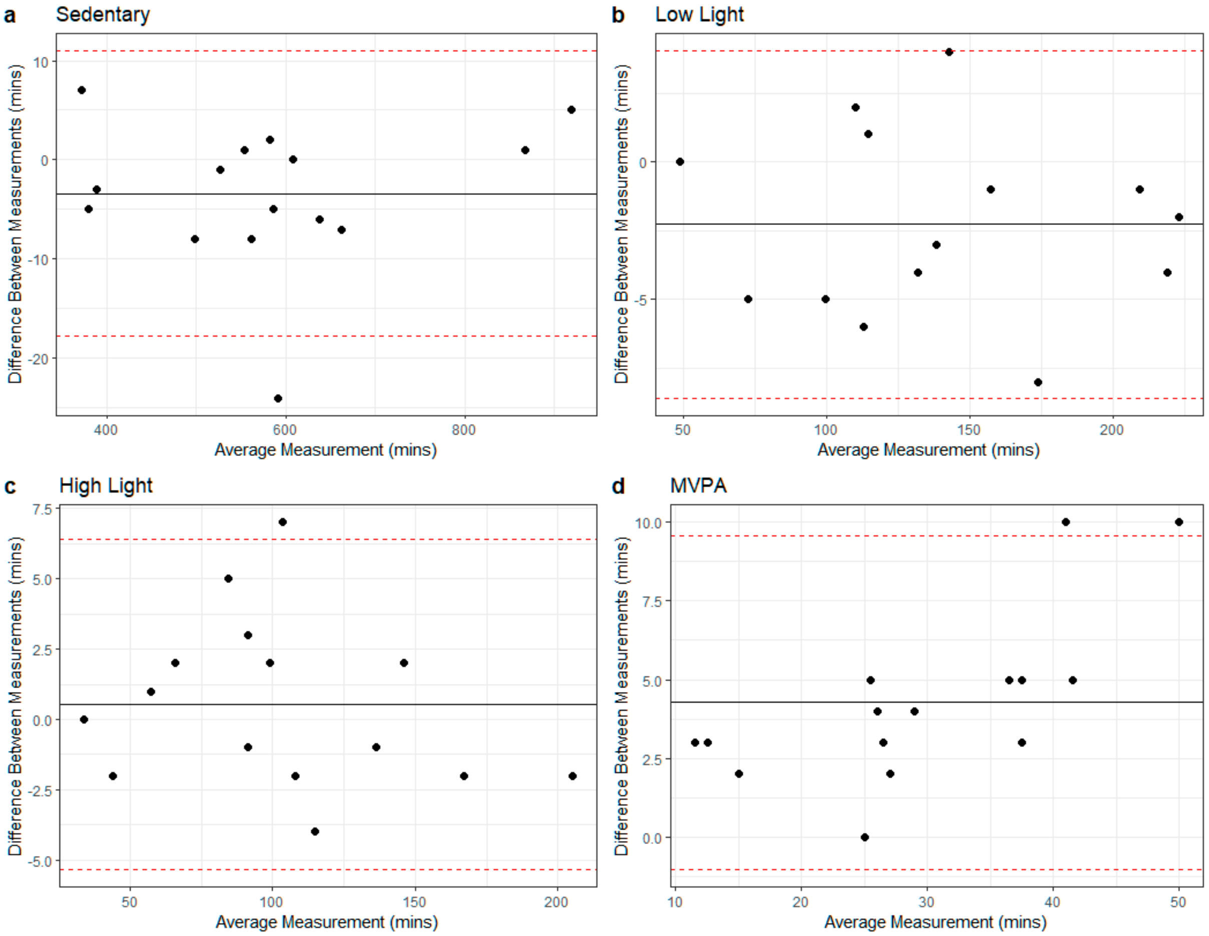

Supplemental Table S1a: Side by Side Comparisons of Wake/Sleep/Non-wear Times-Participant A

Date	Wake Time		Sleep Time		Non-wear time		Wearing time		Decision on inclusion		Reason for exclusion	
	ActiLife	R	ActiLife	R	ActiLife	R	ActiLife	R	ActiLife	R	ActiLife	R
**11/30/2021**	7:00 AM	7:07:00	9:15 PM	21:16:00	9.75	0	14.25	14.15	Include	Exclude		1st day of study
**12/1/2021**	6:45 AM	6:46:00	9:00 PM	21:00:00	9.75	0	14.25	14.23	Include	Include		
**12/2/2021**	7:00 AM	6:54:00	8:15 PM	20:20:00	10.75	0	13.25	13.43	Include	Include		
**12/3/2021**	7:00 AM	7:02:00	11:00 PM	23:03:00	8	0	16	16.02	Include	Include		
**12/4/2021**	7:30 AM	7:22:00	10:00 PM	21:52:00	9.5	0	14.5	14.5	Include	Include		
**12/5/2021**	8:00 AM	8:06:00	10:15 PM	22:12:00	9.75	0	14.25	14.1	Include	Include		
**12/6/2021**	7:00 AM	7:01:00	9:45 PM	21:48:00	9.25	0	14.75	14.78	Include	Include		
**12/7/2021**	7:30 AM	7:37:00	9:45 PM	21:40:00	9.75	0	14.25	14.05	Include	Include		
**12/8/2021**	7:00 AM	7:02:00	9:00 PM	20:51:00	10	0	14	13.82	Include	Include		
**12/9/2021**	6:45 AM	6:51:00	9:30 PM	21:23:00	9.25	0	14.75	14.53	Include	Include		
**12/10/2021**	7:30 AM	7:25:00	10:00 PM	22:04:00	9.5	0	14.5	14.65	Include	Include		
**12/11/2021**	8:30 AM	8:31:00	11:00 PM	22:53:00	9.5	0	14.5	14.37	Include	Include		
**12/12/2021**	5:30 AM	5:32:00	7:30 PM	20:32:00	10	0	14	15	Include	Include		
**12/13/2021**	7:15 AM	7:08:00	10:00 PM	22:05:00	9.25	0	14.75	14.95	Include	Include		
**12/14/2021**	7:15 AM	7:20:00	9:30 PM	21:24:00	9.75	0	14.25	14.07	Include	Include		
**12/15/2021**	7:15 AM	5:24:00	8:30 PM	20:36:00	10.75	1.5	13.25	13.7	Include	Include		
**12/16/2021**	7:30 AM	7:26:00	8:45 PM	20:43:00	10.75	0	13.25	13.28	Include	Include		
**12/17/2021**	7:15 AM	7:16:00	8:30 PM	20:31:00	10.75	0	13.25	13.25	Include	Include		
**12/18/2021**	7:30 AM	7:34:00	10:45 PM	22:40:00	10.75	0	15.25	15.1	Include	Include		
**12/19/2021**	7:00 AM	7:01:00	8:45 PM	20:49:00	10.25	0	13.75	13.8	Include	Include		
**12/20/2021**	7:15 AM	7:10:00	9:15 PM	21:13:00	10	0	14	14.05	Include	Include		
**12/21/2021**		7:03:00		19:27:00		10.5		1.9		Exclude		Day 21; wear time < 10 hours
**12/22/2021**		7:17:00		14:07:00		0		6.83		Exclude		> Day 21; wear time < 10 hours

Supplemental Table S1b: Side by Side Comparisons of Wake/Sleep/Non-wear Times-Participant C

Date	Wake Time		Sleep Time		Non-wear time		Wearing time		Decision on inclusion		Reason for exclusion	
	ActiLife	R	ActiLife	R	ActiLife	R	ActiLife	R	ActiLife	R	ActiLife	R
**9/3/2022**	9:45 AM	9:47:00	9:15 PM	21:09:00	12.5	0.75	11.5	10.62	Include	Exclude		First day of study
**9/4/2022**	8:30 AM	8:23:00	9:00 PM	20:59:00	11.5	0.75	12.5	11.85	Include	Include		
**9/5/2022**	9:00 AM	8:55:00	11:15 PM	23:08:00	9.75	0	14.25	14.22	Include	Include		
**9/6/2022**	11:15 AM	11:11:00	11:59 PM	23:51:00	11.27	0	12.73	12.67	Include	Include		
**9/7/2022**	10:15 AM	10:15:00	11:15 PM	23:13:00	11	0	13	12.97	Include	Include		
**9/8/2022**	12:30 AM	12:34:00	9:00 PM	21:05:00	14.5	0	9.5	8.52	Exclude	Exclude	10> hours of wear	Wear time < 10 hours
**9/9/2022**	7:15 AM	7:13:00	11:30 PM	23:25:00	7.75	0.75	16.25	15.45	Include	Include		
**9/10/2022**	9:45 AM	9:49:00	9:15 PM	23:37:00	12.5	0.75	11.5	13.05	Include	Include		
**9/11/2022**	9:15 AM	9:19:00	11:59 PM	23:54:00	9.27	2.25	14.73	12.33	Include	Include		
**9/12/2022**	2:45 PM	14:39:00	11:45 PM	23:40:00	15	0	9	9.02	Exclude	Exclude	10> hours of wear	Wear time < 10 hours
**9/13/2022**	5:15 AM	5:10:00	11:45 PM	23:48:00	5.5	5.25	18.5	13.38	Include	Include		
**9/14/2022**	9:45 AM	9:52:00	10:15 PM	22:11:00	11.5	2.25	12.5	10.07	Include	Include		
**9/15/2022**	8:00 AM	7:55:00	11:59 PM	23:58:00	8.02	9.75	15.98	6.3	Include	Exclude		Wear time < 10 hours
**9/16/2022**	9:30 AM	9:24:00	10:15 PM	22:13:00	11.25	3.75	12.75	9.07	Include	Exclude		Wear time < 10 hours
**9/17/2022**	10:45 AM	7:51:00	11:30 PM	23:24:00	11.25	6.75	12.75	8.8	Exclude	Exclude	large period of time without movement	Wear time < 10 hours
**9/18/2022**	11:45 AM	11:52:00	10:30 PM	22:22:00	13.25	7.5	10.75	3	Exclude	Exclude	large period of time without movement	Wear time < 10 hours
**9/19/2022**	8:45 AM	8:43:00	11:15 PM	23:09:00	9.5	3.75	14.5	10.68	Include	Include		
**9/20/2022**	9:30 AM	9:19:00	11:45 PM	23:59:00	9.75	2.25	14.25	12.42	Include	Include		
**9/21/2022**	8:30 AM	8:23:00	11:59 PM	23:59:00	8.52	0.75	15.48	14.85	Include	Include		
**9/22/2022**	10:30 AM	10:27:00	11:45 PM	23:41:00	10.75	0	13.25	13.23	Include	Include		
**9/23/2022**		20:57:00		20:57:00		0		0	Exclude	Exclude	10> hours of wear	Wear time < 10 hours

**9/24/2022**		10:35:00		23:46:00		6		7.18	Exclude	Exclude	transit day	Day 21; wear time < 10 hours
**9/25/2022**		23:02:00		23:07:00		0		0.08	Exclude	Exclude	10> hours of wear	> Day 21; wear time < 10 hours
**9/26/2022**		4:00:00		15:58:00		4.5		7.47	Exclude	Exclude	transit day	> Day 21; wear time < 10 hours
**9/27/2022**		13:51:00		17:48:00		0		3.95		Exclude		> Day 21; wear time < 10 hours
**9/28/2022**										Exclude		> Day 21; data not available
**9/29/2022**		12:23:00		15:31:00		0		3.13		Exclude		> Day 21; wear time < 10 hours
**9/30/2022**		9:46:00		9:57:00		0		0.18		Exclude		> Day 21; wear time < 10 hours
